# Ion release and recharge from a fissure sealant containing amorphous calcium phosphate

**DOI:** 10.1371/journal.pone.0241272

**Published:** 2020-11-05

**Authors:** Mahtab Memarpour, Neda Afzali Baghdadabadi, Azade Rafiee, Mehrdad Vossoughi

**Affiliations:** 1 Oral and Dental Disease Research Center, Department of Pediatric Dentistry, School of Dentistry, Shiraz University of Medical Sciences, Shiraz, Iran; 2 Oral and Dental Disease Research Center, Department of Dental Public Health, School of Dentistry, Shiraz University of Medical Sciences, Shiraz, Iran; Universitat Bern, SWITZERLAND

## Abstract

To assess- the release of calcium and phosphate ions from a fissure sealant containing amorphous calcium phosphate (ACP), and to determine the re-release capacity of these ions when charged with a solution containing casein phosphopeptide-amorphous calcium phosphate (CPP-ACP). Nine blocks of ACP resin-based sealant were prepared and immersed in three solutions at different pH (4.0, 5.5, 7.0), and calcium and phosphate ion release was measured with ion chromatography at 1, 3, 5, 7, 14, 21 and 28 days after immersion. Sixty days after immersion, each block was charged with CPP-ACP solution in three 7-day cycles to investigate the re-release of these ions, which was measured on days 1, 3, and 7. No difference was observed in initial calcium ion release at pH 4.0 and pH 5.5. At both values, ion release was significantly higher than at pH 7.0 (*p*<0.001). Initial phosphate release was significantly different among the three pH values (*p*<0.001). After re-charging the specimens, calcium ion re-release was greater than phosphate ion release. Initial ion release from ACP resin-based sealant was greatest at the lowest pH. Ion release decreased with time. As the number of recharge cycles increased, ion re-release also improved. Phosphate ion re-release required more recharge cycles than calcium ion re-release.

## 1. Introduction

The occlusal surface of newly erupted teeth is an area susceptible to dental caries [1]. One of the most effective methods to prevent tooth caries is using a pit and fissure sealant (FS), which acts as a physical barrier to the penetration of bacteria and fermentable sugars in the deepest parts of tooth pits and fissures [2]. However, several factors may influence the long-term success of FS, such as oral biofilms containing *S*. *mutans*, which has esterase activity that hydrolyzes the ester bonds in materials containing methacrylate [3, 4], or enamel demineralization in some parts of the teeth [5]. For this reason, particles such as fluoride or amorphous calcium phosphate (ACP) are added to FS materials to improve the preventive properties of sealants by increasing enamel remineralization and decreasing subsequent demineralization and secondary caries [6–9].

Materials used in dentistry can be classified as bioinert (passive), bioactive, and bio-responsive based on their interactions with the environment [10]. *Smart materials*, also referred to as responsive materials, are able to change their properties in controlled conditions such as temperature, moisture, pH and light [11, 12]. They can also be modified by forms of mechanical stress such as tensile and compressive stress [10–12]. Ion release from these materials has been measured in some studies [13–20].

Amorphous calcium phosphate acts as an important intermediate product for in vitro and in vivo apatite formation, and was the first product to be used as artificial hydroxyapatite [21]. Its unstable and reactive nature in aqueous media causes the release of calcium and phosphate ions and their transformation into crystalline phases due to microcrystalline growth. This makes it a candidate for tissue repair, remineralization and regeneration [10, 21, 22]. The combination of ACP with restorative materials leads to enhanced remineralization of tooth structures, and provides anticariogenic properties [23, 24]. Amorphous calcium phosphate can be added to restorative materials such as adhesives, resin composites and fissure sealants [3, 25–28]. Bioactive resin composites containing ACP release ions after the dental plaque is exposed to acidic condition. Several studies have shown that changing the pH of dental plaque from a neutral to a cariogenic value (pH 4.0) is less occur in the presence of calcium and phosphate-containing materials [14–17, 28]. However, one study reported that ion release was not affected by pH changes [18].

Aegis^®^ is a light-cured FS containing a urethane dimethacrylate (UDMA) base and a resin base of mono- and dimethacrylate filler; the ACP filler accounts for 38% of its weight ratio [29, 30]. At neutral or high pH values ACP remains stable. However, when the pH drops to 5.8 or lower, calcium and phosphate ions are released from ACP into the saliva, where they act as a buffer and are deposited onto tooth structures as apatite mineral, which is similar to hydroxyapatite found naturally in teeth and bone. This phenomenon helps to change the pH from acidic to neutral, i.e. 7.4 [11, 29, 31]. Thus ACP-containing sealants may help prevent caries in two ways: as a physical barrier due to micromechanical adhesion, and as a mineral ion reservoir thanks to the presence of the ACP filler, which increases enamel remineralization and prevents further demineralization [8, 13, 14, 32].

Some research reported that ion release from smart materials was not sustained in the long term, and diminished with time [19, 20]. Therefore, the recharge capacity of these materials is considered a useful characteristic that allows them to maintain their properties, and thereby enhances their ability to prevent caries in the long term [3]. The recharge capacity of smart materials was investigated in previous studies, which yielded different results depending on the materials and methods used to evaluate recharge ability. Most ACP-containing materials include a commercial resin composite or synthetic materials [3, 25, 26]. Only one earlier study measured calcium and phosphate ion release from Aegis^®^ sealant compared to three laboratory-synthesized pit and fissure sealants. The results showed better ion release from the nano-ACP sealant than from the commercial Aegis^®^ sealant [27]. However, that study provided no information about ion re-release after the sealants were recharged.

Therefore, the objective of the present in vitro study was to measure the release of calcium and phosphate ions from the ACP-containing Aegis^®^ fissure sealant after exposure to neutral pH (pH 7.0) and two acidic conditions (pH 4.0 and pH 5.5). In addition, the calcium and phosphate recharge capacity of the sealant following exposure to a solution containing casein phosphopeptide—amorphous calcium phosphate (CPP-ACP) was determined by ion chromatography. Our hypothesis (H0) was that the release of calcium and phosphate ions from the ACP-containing Aegis^®^ fissure sealant would be similar and lower under the two acidic conditions compared to the neutral pH condition, and would approach zero in neutral pH assays. The null hypothesis was tested against an alternative hypothesis (HA) that differences in ion release would be found between the two acidic pH conditions.

## 2. Materials and methods

The research protocol was approved by the Human Ethics Review Committee of the Faculty of Dentistry, Shiraz University of Medical Sciences.

### 2.1. Sample preparation

According to the previous studies on ion release, a total of nine Aegis^®^ fissure sealant (Bosworth Aegis, Keystone Industries, Gibbstown, NJ, USA) (S1 Table) specimens were prepared [3, 25, 26]. Molds of condensational silicone impression material (Speedex, Coltene, Altstätten, Switzerland) measuring 2×2×12 mm were prepared, then the sealant was inserted into each mold and each 1-mm layer was light cured for 40 s with a halogen light curing unit (Coltolux, Coltene/Whaledent AG, Altstätten, Switzerland) at a power density of 550 mW/cm^2^. Each prepared sealant specimen was removed from the mold and cured for 40 s from a distance of 1 mm perpendicular to the surfaces [3, 25, 26].

Three solutions at different pH were used to simulate various pH conditions as follows:

Group 1 (control) consisted of solutions at pH 7.0 (deionized distilled water, SKG, Ahvaz, Iran).
Group 2 consisted of solutions at pH 5.5 (buffer solution, Vaheb, Ahvaz, Iran).
Group 3 consisted of solutions at pH 4.0 (buffer solution, Merck, Darmstadt, Germany).

The solutions were used to store three samples for each pH value. The specimens were immersed in 16 ml of the solutions to yield a specimen volume per solution of 3 mm^3^/ml, which was similar to the sample volume per solution used in a previous study [26]. During the first week of the study (days 1, 3, 5, and 7) each solution was replaced every 48 h. After day 7 the solutions were replaced weekly. The storage period lasted for 3 weeks. The pH of each immersion solution was monitored in each step of the study. At the beginning of this research, Clinpro^™^ Sealant was used as a control group. Since no ion release was observed in our solutions, this group was excluded. Supporting information (S1 Table) shows the detailed chemical composition of the buffer solutions.

### 2.2. Initial ion release measurement

Initial ion release from the specimens was determined first, to differentiate this variable from ion re-release after sample recharge. To determine the concentration of calcium and phosphate ions in initial release tests, after 24 h of immersion in the three buffer solutions, a volume of 5 ml from each sample was collected and analyzed by ion chromatography (883 Basic IC plus, Metrohm AG, Herisau, Switzerland). In addition, the concentration of calcium and phosphate ions released was measured on days 3, 5, 7, 14, 21 and 28. At each time 5 ml of the solutions from each sample was removed for analysis. The remaining solutions were discarded and the samples were immersed in fresh solutions (dynamic approach) [18, 27]. After solution samples were obtained on day 28, the sealant specimens were immersed in the solutions for 1 month to ensure no further ion release on days 58 and 60 of the study. On day 60, measurements revealed no further ion release.

### 2.3. Recharging solution

To prepare the recharging solutions, CPP-ACP paste (Tooth Mousse, GC, Tokyo, Japan) (S1 Table) and deionized water were used at a ratio of 1:3 [33]. Each 5 ml of deionized water was stirred with 1.7 g CPP-ACP in a vortex machine (ZX3 Vortex Mixer, VELP Scientifica, Usmate Velate MB, Italy) at a power setting of 3 for 1 min [26]. Tooth Mousse contains 10% CPP-ACP with 325 mM calcium and 187 mM phosphate [34, 35].

### 2.4. Specimen recharge and re-release

After 60 days, sealant samples were removed from the storage solutions and rinsed with distilled water. These exhausted specimens were used for ion recharge. After the recharge solution was prepared, each specimen was immersed in 5 ml of this solution and stirred gently with a vortex machine for 1 min. Next the specimen was left in the recharging solution for 30 min to simulate clinical conditions, i.e. instructions that the mouth should not be rinsed for at least half an hour after application [33]. Then the sample was rinsed with 10 ml deionized water to remove any loosely attached deposits and ions on the surfaces. Each sealant sample was recharged twice at the beginning and end of the day in each cycle (at 9 AM and 5 PM) to simulate mouth usage in the morning and evening [3, 25, 26]. Next, the specimens were transferred to individual dishes with 16 ml fresh buffer solution (pH 4.0, pH 5.5, pH 7.0). On days 1, 3, and 7, as in the first step of the study, 5 ml of solution was collected from each dish to measure the concentration of re-released calcium and phosphate ions. At each time the blocks were immersed in fresh buffer solutions. To test the influence of increasing numbers of recharge cycles, ion recharge and re-release was repeated in three cycles during 3 weeks (21 days). In other words, the specimens were recharged after day 7, and then tested for ion re-release in cycle 2 (day 14). The same procedure was repeated to measure ion release in cycle 3 (day 21).

### 2.5. Statistical analysis

The data were analyzed with SPSS version 22.0 (IBM SPSS, Chicago IL, USA). To evaluate the effect of pH and immersion time on initial ion release, two-way analysis of variance (ANOVA) was used. To evaluate the effect of the number of recharge cycles, pH and immersion time on ion recharge and re-release, three-way ANOVA was used. If there was a significant interaction effect between variables, subgroup analysis was done based on one-way ANOVA and the Duncan post hoc test. The level of significance was set at 0.05.

## 3. Results

### 3.1. Initial calcium and phosphate ion release

Two-way ANOVA of the data for initial ion release indicated a significant interaction effect between time (day) and pH for both Ca^2+^ and PO4^3−^ (*p*<0.001). Therefore, subgroup analysis was done with one-way ANOVA and the Duncan test.

**Fig 1:**
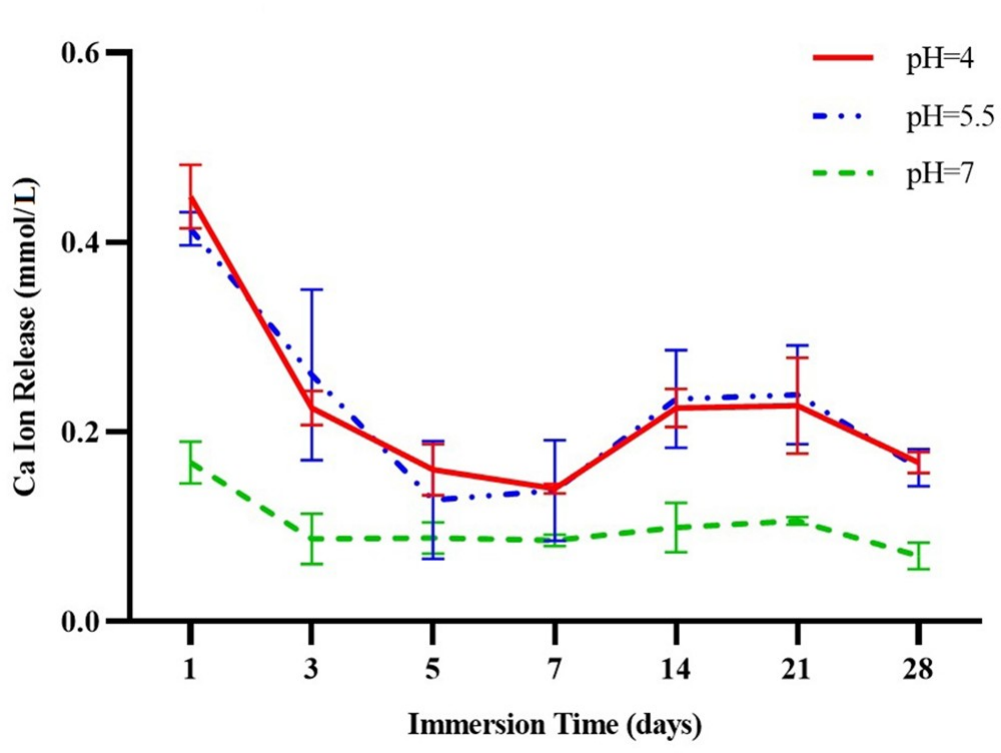
Calcium ion release (millimoles per liter) at three different pH values before recharge (pH 4.0, pH 5.5, pH 7.0).

Table 1 and Fig 1 show the mean calcium ion release at three different pH values (pH 7.0, pH 5.5, pH 4.0) from day 1 to day 28. There was no significant difference between pH 4.0 and pH 5.5. Ca^2+^ release at pH 7.0 on days 1, 3, 14, 21 and 28 was significantly lower than at the other two pH values. However, this difference was not statistically significant for day 5 (*p* = 0.170) and day 7 (*p* = 0.127). The ratios of calcium ion release at pH 4.0 relative to pH 7.0 were between 1.6 and 2.6 on study days 1 to 28.

**Table 1:** Mean and standard deviation for calcium ion release from three blocks (millimoles per liter) at three different pH values.

Day	Mean±SD (mmol/L)			*p* value*
	pH 4.0	pH 5.5	pH 7.0	
**1**	0.448±0.034 ^A,a^	0.414±0.017 ^A,a^	0.168±0.022 ^B,a^	<0.001
**3**	0.225±0.018 ^A,b^	0.260±0.090 ^A,b^	0.087±0.026 ^B,bc^	0.019
**5**	0.161±0.027 ^A,c^	0.128±0.062 ^A,c^	0.088±0.016 ^A,bc^	0.170
**7**	0.141±0.005 ^A,c^	0.138±0.053 ^A,cd^	0.086±0.006 ^A,bc^	0.127
**14**	0.225±0.020 ^A,b^	0.235±0.052 ^A,bc^	0.099±0.026 ^B,bc^	0.006
**21**	0.228±0.050 ^A,b^	0.239±0.052 ^A,bd^	0.107±0.004 ^B,b^	0.015
**28**	0.168±0.011 ^A,c^	0.162±0.019 ^A,bd^	0.069±0.014 ^B,c^	<0.001
***p* value**^†^	<0.001	<0.001	<0.001	

*: One-way ANOVA F test to compare different pH values.

^†^: One-way ANOVA F test to compare different days.

Similar uppercase letters indicate lack of significant differences among three different pH values. Three pairwise comparisons were done in each row using Duncan’s multiple comparison test only in the case of a significant result with one-way ANOVA.

Similar lowercase letters indicate lack of significant differences among different days. Twenty one pairwise comparisons were done in each column using Duncan’s multiple comparison test only in the case of a significant result with one-way ANOVA.

**Fig 2:**
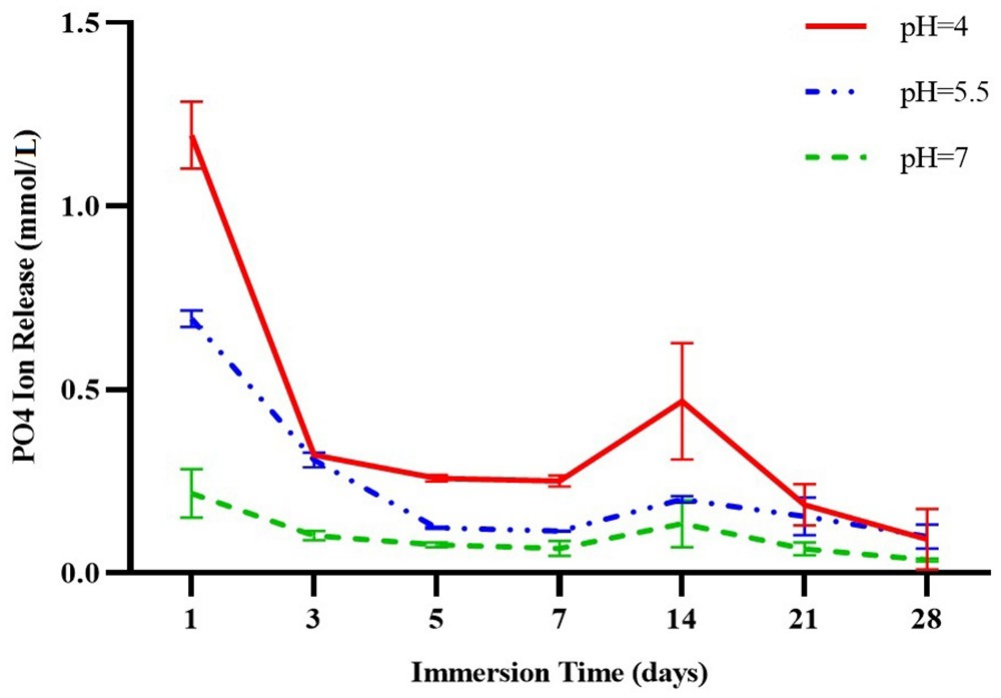
Phosphate ion release (millimoles per liter) at three different pH values before recharge (pH 4.0, pH 5.5, pH 7.0).

Table 2 and Fig 2 show the initial mean PO4^3−^ release at three different pH values (pH 7.0, pH 5.5, pH 4.0) from day 1 to day 28. Comparisons of phosphate ion release between the three pH values on each day showed a statistically significant difference on all days except day 28, when the differences between release at the three pH values were not significant (*p* = 0.327). The ratios of phosphate ion release at pH 4.0 relative to pH 7.0 were between 2.5 and 5 on study days 1 to 28.

**Table 2:** Mean and standard deviation for phosphate ion release from three blocks (millimoles per liter) at three different pH values.

Day	Mean±SD (mmol/L)			*p* value*
	pH 4.0	pH 5.5	pH 7.0	
**1**	1.194±0.091 ^A,a^	0.694 ±0.023 ^B,a^	0.217±0.066 ^C,a^	<0.001
**3**	0.322±0.001 ^A,bc^	0.308±0.021 ^A,b^	0.102 ±0.013 ^B,bc^	<0.001
**5**	0.258±0.001 ^A,bd^	0.123±0.003 ^B,c^	0.077±0.006 ^C,bc^	<0.001
**7**	0.251±0.015 ^A,bd^	0.115±0.002 ^B,d^	0.067±0.019 ^C,bc^	0.001
**14**	0.468±0.015 ^A,c^	0.200±0.001 ^B,e^	0.135±0.064 ^B,b^	0.038
**21**	0.186±0.056 ^A,bd^	0.155±0.052 ^AB,cde^	0.066±0.018 ^B,bc^	0.041
**28**	0.091±0.083 ^A,d^	0.099±0.032 ^A,cd^	0.036±0.003 ^A,c^	0.327
***p* value**^†^	<0.001	<0.001	<0.001	

*: One-way ANOVA F test to compare different pH values.

^†^: One-way ANOVA F test to compare different days.

Similar uppercase letters indicate lack of significant differences among three different pH values. Three pairwise comparisons were done in each row using Duncan’s multiple comparison test only in the case of a significant result with one-way ANOVA.

Similar lowercase letters indicate lack of significant differences among different days. Twenty one pairwise comparisons were done in each column using Duncan’s multiple comparison test only in the case of a significant result with one-way ANOVA.

Comparisons of ion release on different days at each pH showed that on day 1, calcium and phosphate ion release was significantly greater than on any other day (*p*<0.001). In the first week, ion release decreased with time, and some decreases between successive days were significant. Between days 7 and 14, a small increase was observed in both calcium and phosphate ion release in all groups, which was due to the longer sampling interval compared to the first week of the study.

Calcium ion release at all three pH values showed no significant difference between days 14 and 21. On day 28, ion release decreased compared to day 21 at all three pH values. From day 14, phosphate ion release remained uniform at pH 5.5 and pH 7.0. At pH 4.0, phosphate ion release was significantly greater than at the other two values; however, ion release decreased thereafter and on day 28, it reached the same values as recorded at pH 5.5 and pH 7.0.

For more information about the cumulative calcium and phosphate ion release see S1 and S2 Figs.

### 3.2. Recharge and re-release

**Fig 3:**
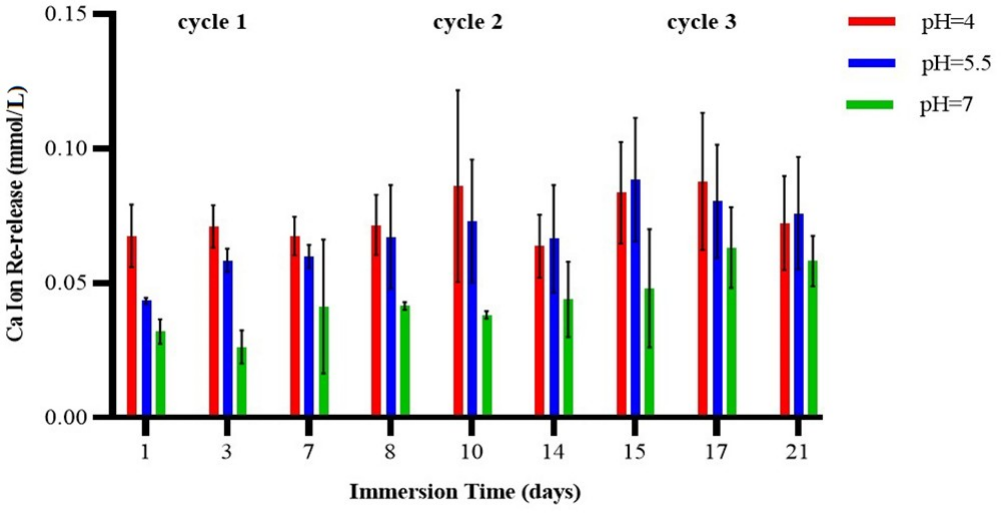
Calcium ion re-release (millimoles per liter) at three different pH values and different days of the 7-day cycle (pH 4.0, pH 5.5, pH 7.0).

Three-way ANOVA of the data for calcium ion re-release after the samples were recharged indicated no significant interaction effects between cycle, pH, and day (*p* = 0.901), between cycle and pH (*p* = 0.696), between cycle and day (*p* = 0.708), or between pH and day (*p* = 0.543). When the effect of time and cycle were controlled for, a significant difference was observed between the three pH values (*p*<0.001). The Duncan test indicated that mean calcium ion release at pH 4.0 was significantly greater than at pH 5.5 or pH 7.0. In addition, when time and pH were controlled for, a significant difference was observed between all three cycles (*p*<0.001), such that ion release was higher in cycle 3 compared to cycle 2, and was higher in cycle 2 compared to cycle 1. When the effect of pH and cycle was controlled for, no significant difference was observed between the three days (*p* = 0.557) (Table 3 and Fig 3).

**Table 3:** Mean and standard deviation for calcium ion re-release from three blocks (millimoles per liter) at three different pH values and different days of the 7-day cycle.

Effect	Category	Mean±SD (mmol/L)	*p* value*
**pH**	4.0	0.0746±0.0176 ^A^	<0.001
	5.5	0.0681±0.0194 ^A^	
	7.0	0.0436±0.0159 ^B^	
**Cycle**	1	0.0519±0.0181 ^A^	<0.001
	2	0.0613±0.0219 ^B^	
	3	0.0731±0.0212 ^C^	
**Day**	1	0.0604±0.0228 ^A^	0.557
	3	0.0649±0.0257 ^A^	
	7	0.0610±0.0174 ^A^	

Three-way ANOVA detected no significant interaction effect between pH, cycle or day.

*: Three-way ANOVA F test to test main effects.

The same letters in each category indicate lack of significant differences. If the *p* value for main effect was significant, 3 pairwise multiple comparisons were done with Duncan’s multiple comparison test.

**Fig 4:**
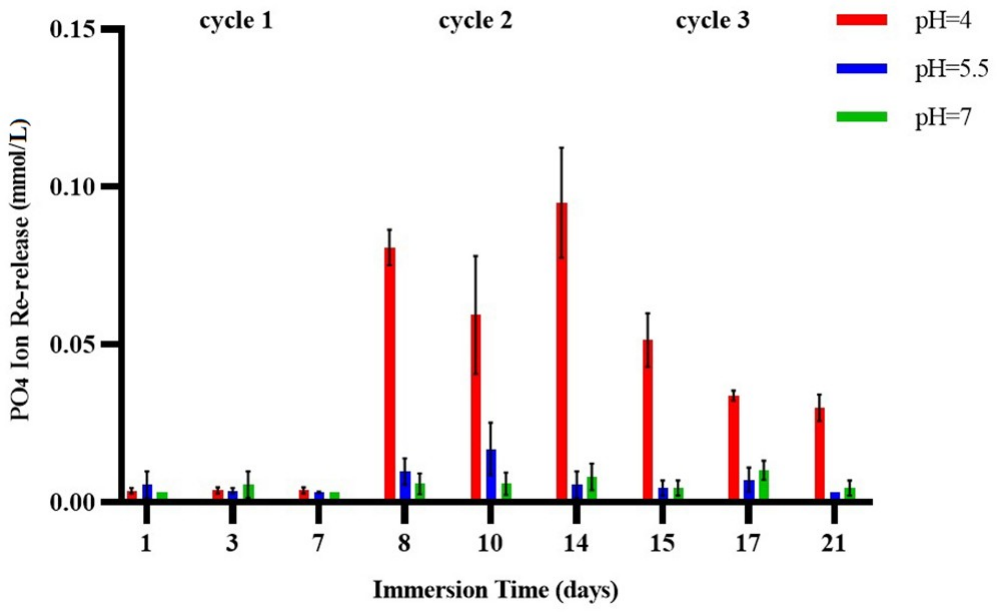
Phosphate ion re-release (millimoles per liter) at three different pH values and different days of the 7-day cycle (pH 4.0, pH 5.5, pH 7.0).

Three-way ANOVA of the data for phosphate ion re-release after the samples were recharged indicated significant interaction effects between cycle, pH and time (*p*<0.001), cycle and pH (*p*<0.001), cycle and time (*p* = 0.012), and between pH and time (*p*<0.001). Ion release on all days of cycle 1 showed no significant differences among the three pH values (*p*>0.05). However, on all days of cycles 2 and 3, average ion release at pH 4.0 was significantly higher than at the other two pH values. At pH 5.5 and pH 7.0, no significant difference was observed between cycles in mean ion release on any of the days. At pH 4.0 on all days, mean ion release was higher in cycle 2 than in cycle 3, and was higher in cycle 3 than in cycle 1. Except for cycle 3 at pH 4.0, no significant difference was observed in ion release on any of the three days. In cycle 3 at pH 4.0, mean ion release was higher on day 1 compared to days 3 and 7 (Table 4 and Fig 4).

**Table 4:** Mean and standard deviation for phosphate ion re-release from three blocks (millimoles per liter) at three different pH values and different days of the 7-day cycle.

Cycle	Day	Mean±SD (mmol/L)		
		pH 4.0	pH 5.5	pH 7.0
**1**	**1**	0.0037±0.0009 ^A,a^	0.0056±0.0042 ^A,a^	0.0032±0.0000 ^A,a^
	**3**	0.0038±0.0010 ^A,a^	0.0037±0.0009 ^A,a^	0.0056±0.0042 ^A,a^
	**7**	0.0038±0.0010 ^A,a^	0.0033±0.0002 ^A,a^	0.0032±0.0000 ^A,a^
**2**	**1**	0.0808±0.0056 ^A,b^	0.0098±0.0042 ^B,a^	0.0059±0.0033 ^B,a^
	**3**	0.0594±0.0187 ^A,b^	0.0170±0.0083 ^B,a^	0.0060±0.0035 ^B,a^
	**7**	0.0950±0.0174 ^A,b^	0.0056±0.0042 ^B,a^	0.0081±0.0042 ^B,a^
**3**	**1**	0.0515±0.0080 ^A,c^	0.0046±0.0024 ^B,a^	0.0046±0.0024 ^B,a^
	**3**	0.0339±0.0016 ^A,d^	0.0072±0.0039 ^B,a^	0.0102±0.0030 ^B,a^
	**7**	0.0300±0.0042 ^A,d^	0.0032± 0.0000 ^B,a^	0.0046±0.0024 ^B,a^

Three-way ANOVA detected significant interaction effects between pH, cycles and days.

Similar uppercase letters indicate lack of significant differences among three different pH values. Three pairwise comparisons were done in each row with Duncan’s multiple comparison test.

Similar lowercase letters indicate lack of significant differences among different days and different cycles. Three pairwise comparisons were done to compare both cycles and days in each column with Duncan’s multiple comparison test.

## 4. Discussion

The results of the present study show that a lower pH environment led to enhanced release of calcium and phosphate ions. This finding is in accordance with previous studies of the effect of reducing pH on ion release from calcium- and phosphate-containing materials [15–18]. In addition, the present study found no significant difference between mean calcium ion release at pH 4.0 and pH 5.5, which was in accordance with our null hypothesis. However, ion release at these values was considerably greater than at pH 7.0. As claimed by the Aegis^®^ sealant manufacturer, calcium and phosphate ions are released when the pH decreases to 5.8 [29]. Although our results show that ion release occurred at pH 7.0, the values were significantly lower than at the two other pH values. Thus, the null hypothesis (H0) was partially rejected.

In the present study, calcium and phosphate ion release at all three pH values declined significantly from day 1 to day 3. The reduced ion release may be directly related to pH. At pH 4.0 (acid condition), a decreasing trend in ion release with a steep slope was seen until day 3, whereas at pH 5.5 the slope of this decrease was gentle up to day 5, due to the lower acidity of the test solution. At pH 7.0, calcium ion release was lower compared to the two lower pH groups, and the rate of release remained stable from day 3 to 21. On day 28, calcium ion release was similar at pH 4.0 and pH 5.5, and was lower than on previous days. Phosphate ion release was lowest on day 28 and was similar at all three pH values. A previous study reported reductions in calcium ion release from a resin composite containing ACP from days 7 to 21, which is consistent with the results of the present study [18]. However, that study also found that mean calcium ion release increased on day 28 [18], which contrasts with the present results. The difference between studies may be due to the composition of the materials investigated, the type of acid used, different environments, solution conditions, and different methods used to measure ion release [28]. For phosphate ion release, that study found the same values from day 14 to 28 [18], in agreement with the results of the present study at pH 5.5 and pH 7.0.

In the present study, mean ion release at pH 4.0 relative to pH 7.0 was between 1.6- and 2.6-fold as high for calcium, and between 2.5- and 5-fold as high for phosphate. However, some studies reported higher values of ion release. Xu et al. measured the amount of ions released from a resin composite with Nano-ACP filler. Their results showed that after 28 days, phosphate and calcium ion release at pH 4.0 was, respectively, 5-fold and 10-fold the amount released at pH 7.0 [16]. In a study by Melo et al. [17], calcium and phosphate ion release from a nanocomposite containing a bisphenol A-glycidyl methacrylate (bis-GMA)-tetraethyleneglycol dimethacrylate (TEGDMA) base and ACP filler was greater than in the present study. This may be due to the difference in the chemical structure of the materials used in their study, and to differences in the volume ratio between the specimen and immersion medium [28]. Urethane dimethacrylate is a hydrophobic monomer that is more resistant to water absorption and degradation due to its higher molecular weight and fewer binary bonds compared to TEGDMA—characteristics that reduce the possibility of ion release [36, 37].

In the present study, the specimens were kept in the storage solutions for 1 more month after 28 days. On day 57, they were rinsed and placed in fresh solution, and on days 58 and 60 they were tested for calcium and phosphate ion release. On these two days, ion release was undetectable by ion chromatography. After verifying the lack of ion release from the specimens, the recharge phase of each block started in three 7-day cycles [3, 25, 26]. In total, we recorded data for ion release from each block on 9 different days during the release phase (days 1, 3,5, 7, 14, 21, 28, 58 and 60) and on 9 different days during the recharge phase (days 1, 3, 7, 8, 10, 14, 15, 17 and 21).

One of the techniques to recharge exhausted specimens is soaking in a supersaturated solution of similar ionic composition. The most likely recharge mechanism is the space-occupying effect. After initial calcium and phosphate ion release, the sites that were previously occupied by these ions become available to incoming ions from the recharge solution. [3, 25, 26]. Thus, a better recharge capacity would be expected in smart materials with greater initial ion release. In a study by Zhang et al., adhesives containing the hydroxyethyl methacrylate (HEMA) monomer showed higher initial calcium and phosphate ion release, and also showed greater re-release in the recharging phase [3]. However, in another study the authors reported that the difference in composition between the types of ACP-containing composites led to different ion release rates after recharging compared to ion release in the initial stage [26]. In the present study, there was no difference in calcium ion release at pH 4.0 and pH 5.5 in the initial phase or after recharging. In other words, after further calcium ion release in the initial phase of the experiment, more free spaces were created to accept calcium ions in the specimens, and better rechargeability was provided. However, there were differences in phosphate ion release between the initial phase of the study and the recharge phase. Phosphate ion re-release following recharge at pH 4.0 was significantly higher compared to pH 5.5 and pH 7.0, whereas ion release was lower and did not differ significantly at pH 5.5 and pH 7.0. This may be due to the presence of higher levels of calcium than phosphate in the composition of Tooth Mousse [33, 34]. The greater release of calcium than phosphate ions after recharging in the present study was consistent with previous findings [3, 25, 26]. Another explanation for recharged mechanism is the presence of carbonyl groups in the structure of this sealant that make it possible to chelate with calcium and phosphate ions from the exterior environment such as a recharge solution [38]. One of the reasons for this chelation is the ions release from the sealant after being placed in an acidic solution for 48 hours (without recharging).

The present results show that by increasing the number of recharge cycles, ion release can be enhanced. This finding may be due to the cumulative effects of ions after several recharge cycles. In particular, more recharge cycles are required to recover phosphate ion release. Because of the chemical structure of ACP, it is easier to place calcium in its structure than phosphate [18, 39]. Moreover, CPP-ACP solution contains more calcium ions than phosphate, which results in more calcium uptake and consequently more release [40, 41]. Perhaps with longer exposure times, the ability to release phosphate ions would increase. But we found that the potential for recharging the block with phosphate ions was limited because phosphate release in cycle 3 was lower than in cycle 2. The reasons for this finding may be elucidated more fully by doing experiments with more recharge cycles. However, earlier studies reported that increasing the number of cycles had no effect on ion release [3, 25, 26]. This may be related to the different materials used for recharging, and to the methods of ion measurement used in those studies.

Tooth Mousse was selected for the recharge solutions in the present study because it is a common, well known and commercially available product. This makes it easily available for both personal use and for use in experimental research. The total release of calcium and phosphate ions from smart materials depends on several factors, including the type of base material (e.g. bis-GMA, TEGDMA, and UDMA) [35], acidic monomers [42], percentage of calcium and phosphate fillers [43], size and chemical structure of the fillers [16], the type and acidity of the environment where the material is kept [14], the volume ratio between the specimen and immersion medium, and the method of data collection [28].

One of the limitations of the present study was the duration of immersion of the specimens in acidic pH solutions, which was expected to accelerate ion release compared to the neutral pH condition. The oral cavity is exposed to acidic challenge for several hours each day. Despite the long duration of contact between the samples and the acidic environment in the present study, significant release was observed during 28 days. One of the strengths of the present study compared to previous ones was that the solution was replaced after each sampling, and the specimens were immersed in fresh solution. In most previous studies, solution samples were collected and then fresh new solution was added to the remaining solution, and the data were converted to cumulative values. However, no such cumulative effect on ion release occurs in real-life conditions in the oral cavity, because intra-oral conditions change constantly due to diet, mouthwash use, and the buffering effect of saliva. A further consideration is our choice of deionized distilled water for the experiments done at pH 7.0, as in previous studies [13, 44], because it provided a baseline of ion release potential in unstimulated and neutral conditions, rather than a model of normal oral fluid conditions. In addition, because the presence of calcium and phosphate in solutions is a potential confounding factor in the test results, we used deionized water to produce a neutral pH environment, as in previous studies [13, 44, 45]. Because in vitro studies do not reflect actual clinical conditions, additional clinical studies are needed to investigate initial calcium and phosphate ion release at baseline and after interventions that can affect recharge and re-release.

## 5. Conclusion

The initial release of calcium and phosphate ions from the ACP-containing fissure sealant studied here was greater in media with lower pH values. Ion release decreased with time. Overall calcium ion release in the initial phase was lower than phosphate ion release. After recharging, calcium ion re-release was greater than phosphate ion re-release. More recharge cycles were needed to improve phosphate ion re-release compared to calcium ions. As the number of recharge cycles increased, ion re-release also improved.

## Supporting information

S1 Table The compounds in the materials used in the study. (DOCX)

S1 Fig Cumulative calcium ion release at three different pH values before recharge during 28 days. (TIF)

S2 Fig Cumulative phosphate ion release at three different pH values before recharge during 28 days. (TIF)
