# Supplementary material for: Ion release and recharge from a fissure sealant containing amorphous calcium phosphate
Source: PLoS One. 2020 Nov 5;15(11):e0241272. doi: 10.1371/journal.pone.0241272 (PMC7643944; doi:10.1371/journal.pone.0241272)
Supplement: S1 Table — (DOCX) [file pone.0241272.s001.docx]

**S1 Table. The compounds in the materials used in the study.**

| **Manufacturer** | Composition | Material |
| --- | --- | --- |
| Keystone Industries, USA | UDMA, mono-and di-methacrylate resins, TLV-TWA: 15 mg/m^3^ TWA for ACP, N/A for resin | Aegis |
| GC, Japan | Pure water, glycerol, 10% by weight CPP-ACP (325 mM calcium, 187 mM phosphate), D-sorbitol, CMC-Na, propylene glycol, silicon dioxide, titanium dioxide, xylitol, phosphoric acid, flavoring, zinc oxide, sodium saccharin, ethyl p-hydroxybenzoate, magnesium oxide, guar gum, propyl p-hydroxybenzoate, butyl p-hydroxybenzoate | Tooth Mousse |
| Merck, Germany | Citric acid (21 g/L), sodium hydroxide (8.05 g/L), hydrogen chloride (1.97 g/L), Deionized distilled water | Buffer solution pH 4.0 |
| Vaheb, Iran | Citric acid (24.5 g/L), sodium hydroxide (13.97 g/L), hydrogen chloride (2.98 g/L), Deionized distilled water | Buffer solution pH 5.5 |
| SKG, Iran | Deionized distilled water | Solution pH 7.0 |

UDMA=urethane dimethacrylate; TLV-TWA=threshold limit value-time-weighted average; N/A=not available

Final pH was adjusted up to the desired value using sodium hydroxide or hydrogen chloride
