# Supplementary figures and images for: Ion release and recharge from a fissure sealant containing amorphous calcium phosphate
Source: PLoS One. 2020 Nov 5;15(11):e0241272. doi: 10.1371/journal.pone.0241272 (PMC7643944; doi:10.1371/journal.pone.0241272)

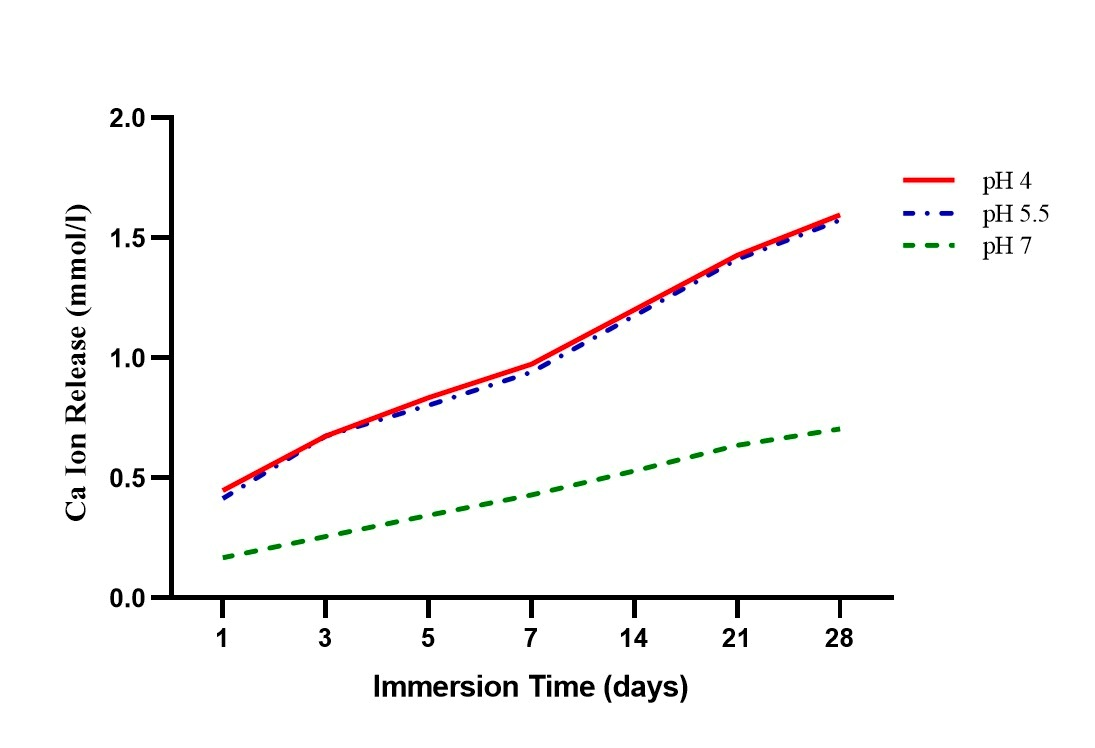

Supplement: S1 Fig — (TIF) [file pone.0241272.s002.tif]

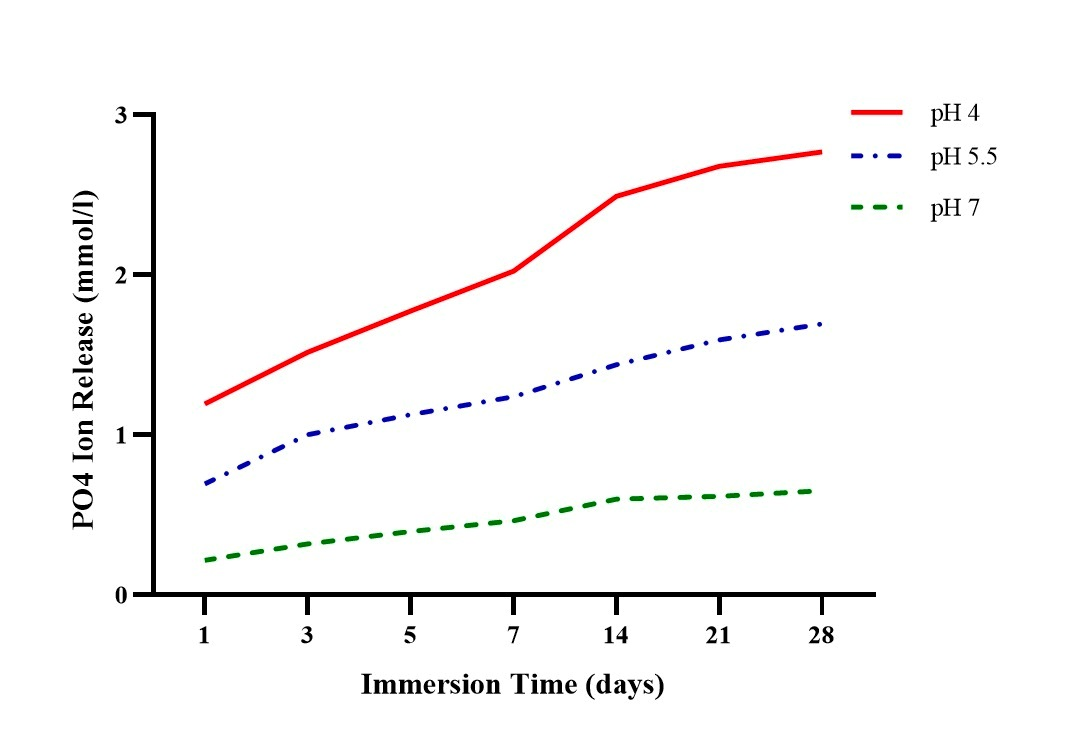

Supplement: S2 Fig — (TIF) [file pone.0241272.s003.tif]
